# Fermentation bed farming improves behavioral expression and stress resistance in geese

**DOI:** 10.3389/fvets.2026.1756186

**Published:** 2026-01-30

**Authors:** Haoyan Li, Jieyu Yang, Jiexing Zhang, Lingbin Zhao, Tong Zhou, Hongsheng Chen, Jiawei Li, Shuai Zhao, Guoan Yin

**Affiliations:** 1 College of Animal Science and Veterinary Medicine, Heilongjiang Bayi Agricultural University, Daqing, Heilongjiang, China; 2 College of Food Science, Heilongjiang Bayi Agricultural University, Daqing, Heilongjiang, China; 3 College of Informatics, Huazhong Agricultural University, Wuhan, Hubei, China; 4 Heilongjiang Provincial Key Laboratory of Exploration and Innovation Utilization of White Goose Germplasm Resources in Cold Region, Daqing, Heilongjiang, China

**Keywords:** behavior, fermentation bed farming, geese, stress resistance, transport stress

## Abstract

**Introduction:**

Conventional livestock farming restricts the expression of natural behaviors and exacerbates animal stress responses. This study investigated the effects of fermentation bed farming, a novel welfare-oriented system, on the behavior and stress resistance of geese.

**Methods:**

240 Northern White Geese (‌*Anser cygnoides*) were randomly assigned to either a flat-floor farming (Ct group) or a fermentation bed farming (Tre group). Data of behavioral patterns and post-transport blood parameters were analyzed.

**Results:**

Compared to the Ct group, the Tre group showed significantly longer durations of sitting, walking, cage pecking, and comforting behaviors, while reduced standing and feather pecking behaviors (*p* < 0.05). Post-transport, serum levels of HSP70, HSP90, CORT, SP, Hpt, IL-2, IL-6, and MDA were significantly lower in the Tre group (*p* < 0.05), whereas levels of IgA, IgG, IgM, IL-4, SOD, and GSH-Px were significantly higher (*p* < 0.05).

**Discussion:**

In conclusion, fermentation bed farming improves behavioral expression and enhances stress resistance in geese.

## 1 Introduction

Environmental enrichment is a technical approach that optimizes captive environments to enhance animals’ biological functions. Its core lies in promoting the improvement of animals’ physiological and behavioral states by increasing environmental complexity (1, 2). Environmental enrichment effectively improves the welfare of captive animals and reduces stress responses by promoting natural behavioral expressions, reducing the occurrence of abnormal and destructive behaviors, alleviating negative emotions, and enhancing immune function (3, 4). For instance, a study by Altan et al. found that early environmental enrichment during the rearing of broiler chickens can significantly reduce their fear levels and effectively enhance their ability to cope with environmental challenges (5). Furthermore, enriched environments not only have a positive impact on behavioral performance but also can improve the meat quality and production performance of captive animals to a certain extent (6). Currently, environmental enrichment for captive animals is mostly achieved by expanding rearing space, enriching social relationships, and adding toys, bedding materials, or other enrichment items.

Fermentation bed farming is an environmentally friendly animal rearing technology that utilizes agricultural wastes such as sawdust, rice husks, and straw as bedding materials, supplemented with microbial consortia to decompose manure. The fermented bedding not only reduces environmental pollution from animal waste but also enriches the environment, thereby improving the living conditions of livestock (7, 8). Studies have shown that this system promotes the expression of comfort behaviors, reduces physical injuries, suppresses abnormal behaviors, and encourages positive behavioral responses in captive animals, while effectively mitigating stress. For instance, Pavlik et al. reported that hens housed in fermentation bed systems displayed lower levels of aggression and reduced corticosterone levels (9).

Transport is an inevitable and critical phase in the management of captive animals, which can induce varying degrees of stress and subsequently affect immunity, behavior, and production performance, thereby compromising animal welfare (10, 11). Studies have shown that transport stress increased blood corticosterone levels, triggers inflammatory responses in the immune system (12), reduces immunoglobulin concentrations (13), and causes oxidative damage (14), potentially leading to mortality (15). Environmental enrichment has been demonstrated to improve both welfare and immune function in animals. Li et al. found that enriched environments mitigated transport-induced immune impairment and enhanced the tolerance of hen to transport stress (16). Similarly, Matur et al. reported that housing laying hens in enriched cages post-transport increased comfort behaviors and reduced aggression compared to conventional cages (17), indicating that environmental enrichment facilitates recovery after experiencing transport stress. Although fermentation bed farming serves as a form of environmental enrichment, its effects on the behavior of geese and its potential to alleviate transport stress remain unexplored. Therefore, this study aims to evaluate the impact of fermentation bed farming on the behavioral expression and stress resistance of geese, in order to determine whether this rearing system can enhance their welfare and tolerance to transport stress.

## 2 Materials and methods

### 2.1 Ethics statement

All experiments were approved by and conducted as per the guidelines of the Institutional Animal Care and Use Committee of Heilongjiang Bayi Agricultural University (DWKJXY2023086).

### 2.2 Animals and experimental design

240 healthy 45 day of age Northern White Geese (*‌Anser cygnoides*) were selected for the trial and randomly allocated into two groups: a flat-floor farming (control group; Ct group) and a fermentation bed raising group (treatment group; Tre group). Each group contained 120 birds, with 6 replicates per group and 20 birds per replicate (female:male = 1:1). The Ct group was raised in a conventional flat-floor system with no bedding material, but merely a layer of sandy soil. While the Tre group was housed on fermentation bed (The bedding consisted of a 40-cm base layer of crushed corn straw and a 5-cm upper layer of rice hulls, onto the surface of which probiotics were uniformly sprayed. The probiotics were sourced from the Research Team of Cold-region Agricultural Waste Bioconversion and High-quality Agricultural Technology Development, Northeast Agricultural University). A 9-week feeding trial was conducted. Each replicate was housed in a pen measuring 3.5 m × 4 m, equipped with an automatic drinking water system and a feed trough. The diet was provided by Daqing Hefeng Co., Ltd. (Daqing, Heilongjiang, China). Its nutritional composition was as follows: crude protein ≥ 16.0%, methionine 0.3–0.9%, crude fiber ≤ 7.0%, crude ash ≤ 12.0%, moisture 12.0%, NaCl 0.3–1.2%, calcium 0.4–1.5%, and total phosphorus ≥0.3%. All animals had *ad libitum* access to feed and water. The housing environment maintained natural ventilation and lighting. Vaccinations were administered in accordance with a standardized immunization protocol following commercial farming practices. During the feeding period, the litter height was regularly monitored. The litter was replenished whenever consumption exceeded 40% of the initial volume. A transportation stress trial was conducted after the 9-week rearing period. Before transport, the geese were fasted for 10 h with *ad libitum* access to water. The total transportation duration was 3 h. Upon completion of transport, one individual was randomly selected from each replicate for euthanasia (Geese were subjected to euthanasia by decapitation performed by professional slaughterers, in strict accordance with the criteria outlined in the AVMA Guidelines for the Euthanasia of Animals of 2020 Edition) and blood collection, to give a final sample size of six geese per group. Blood samples were collected into 10 mL centrifuge tubes and allowed to clot at room temperature. Subsequently, the samples were centrifuged at 2,500 rpm for 10 min. The serum was collected and stored at −80 °C for subsequent analysis of serum parameters.

### 2.3 Behavioral observations

Before the commencement of this trial, Twenty-four geese (12 from the Ct group and 12 from the Tre group, with 2 randomly selected from each replicate) were marked with specialized animal-safe wax crayons to facilitate subsequent behavioral observations. The behavior of the geese was recorded continuously for 3 days during the eighth week of the trial period using video surveillance equipment (AUX-7780, Jovision Technology Co., Ltd., Jinan, China). The cameras were fixed on the pen rails to ensure complete coverage of all replicate pens and the behavior of the experimental geese could be clearly recorded. Definitions of the recorded behaviors are presented in Table 1.

**Table 1:** Categories and definitions of behaviors.

Behaviors		Definitions
State behavior	Feeding	Located next to feeder, head over food or picking food
	Sitting	Chest-on-ground behavior
	Walking	Walking, running, jumping or flying on the ground
	Standing	Stand with your legs on the ground
Event behavior	Drinking	Pecking at the water in the drinker with the beak
	Cage pecking	Peck with beak at surrounding objects
	Preening	Use the beak to gently rub, ruffle, and comb its feathers or use the feet to
	Comforting	Body shaking, tail shaking, wing lifting behavior
	Feather pecking	Pecking or pulling the feathers of other individuals

Following the video recording, all data were stored on portable hard drives and analyzed by a single observer to ensure consistency. Based on diurnal activity patterns and periods of high behavioral intensity, observation periods were selected twice daily (9:00–12:00, 13:00–16:00). State behaviors (including feeding, lying, walking, and standing) were quantified using scan sampling at one second interval during the three-day observation period. The duration of each state behavior was calculated as a percentage of the total observation time. Event behaviors (including drinking, object pecking, preening, comfort behaviors, and feather pecking) were recorded using continuous observation. Each occurrence of an event behavior was counted as one incident, and the total frequency of each event behavior was summarized.

### 2.4 ELISA for serum indicators

Serum concentrations of corticosterone (CORT), substance P (SP), haptoglobin (HP), immunoglobulins (IgA, IgG, IgM), and interleukins (IL-2, IL-4, IL-6) in Northern White Geese were measured using commercial ELISA kits (Shanghai Enzyme Link Biotechnology Co., Ltd., China). The levels of malondialdehyde (MDA), glutathione peroxidase (GSH-Px), and superoxide dismutase (SOD) in goose serum were determined using biochemical assay kits (Suzhou Grace Biotechnology Co., Ltd., China). All procedures were strictly performed according to the manufacturer’s instructions.

### 2.5 Statistical analysis

Statistical analysis was performed using SPSS 26.0. The normality of data was assessed using the Shapiro–Wilk test. Independent t-tests were used for normally distributed data, and the Mann–Whitney U test was used for non-normally distributed data. All data are presented as mean ± SEM. Significance was defined as *p < 0.05, **p < 0.01, and ***p < 0.001 compared to the control group; ‘ns’ indicates not significant.

## 3 Results

### 3.1 Effects of fermentation bed farming on the behaviors of northern white geese

**Figure 1:**
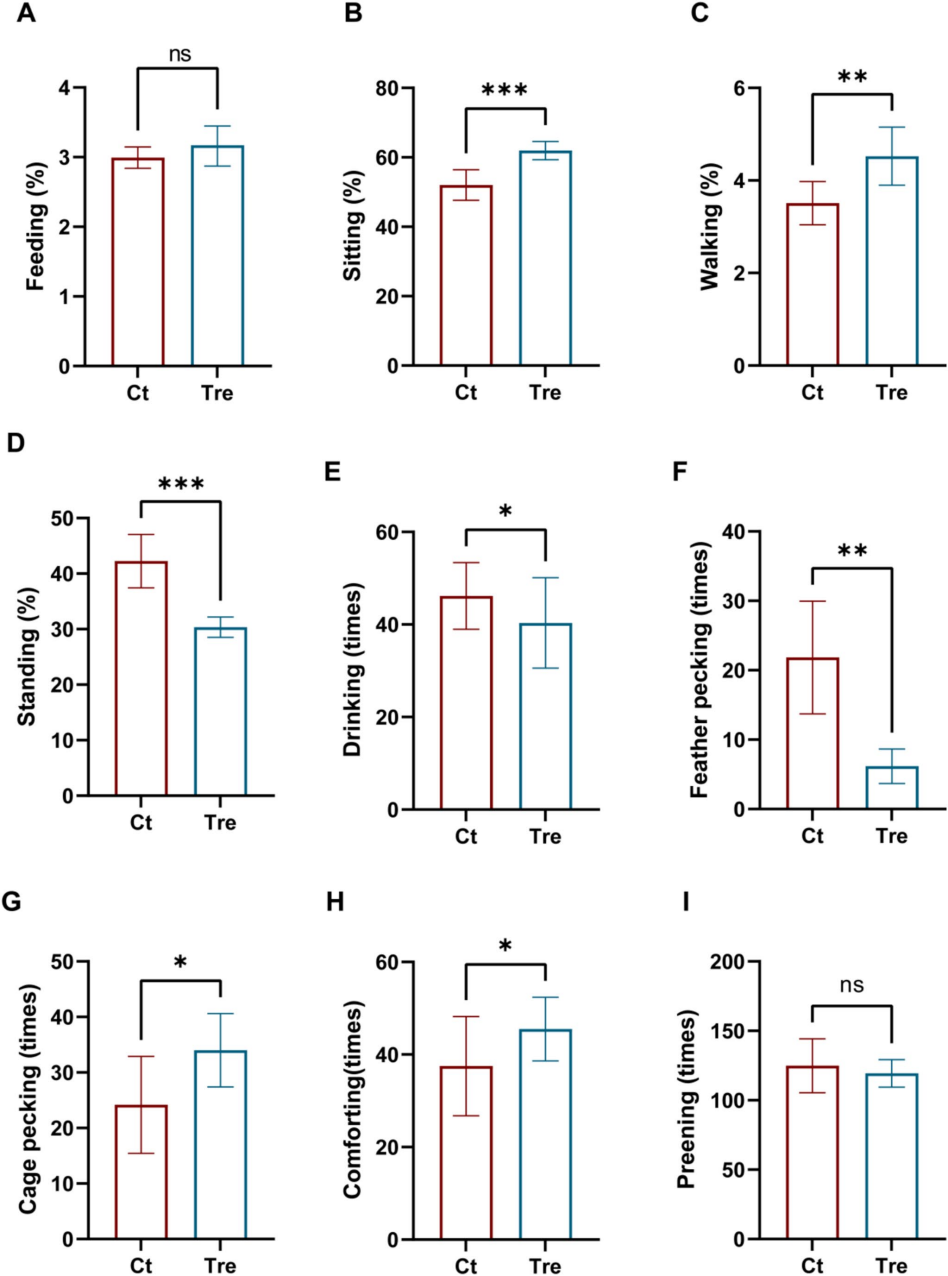
Effects of fermentation bed farming on the behavior of geese **(A–I)**. Ct: control group; Tre: fermentation bed group. All data are presented as mean ± SEM. Significance was defined as *p < 0.05, **p < 0.01, and ***p < 0.001 compared to the control group; ‘ns’ indicates not significant.

The effects of fermentation bed farming on the behaviors of geese are shown in Figure 1. Compared to the Ct group, geese in the Tre group showed a significant increase in sitting, walking, cage pecking, and comforting behaviors (*p* < 0.05), but a significant decrease in standing and feather pecking behaviors (*p* < 0.05). No significant differences were observed in feeding, drinking, and preening behaviors between the two groups (*p* > 0.05).

### 3.2 Effects of fermentation bed farming on serum HSP70 and HSP90 levels in northern white geese following transportation stress

**Figure 2:**
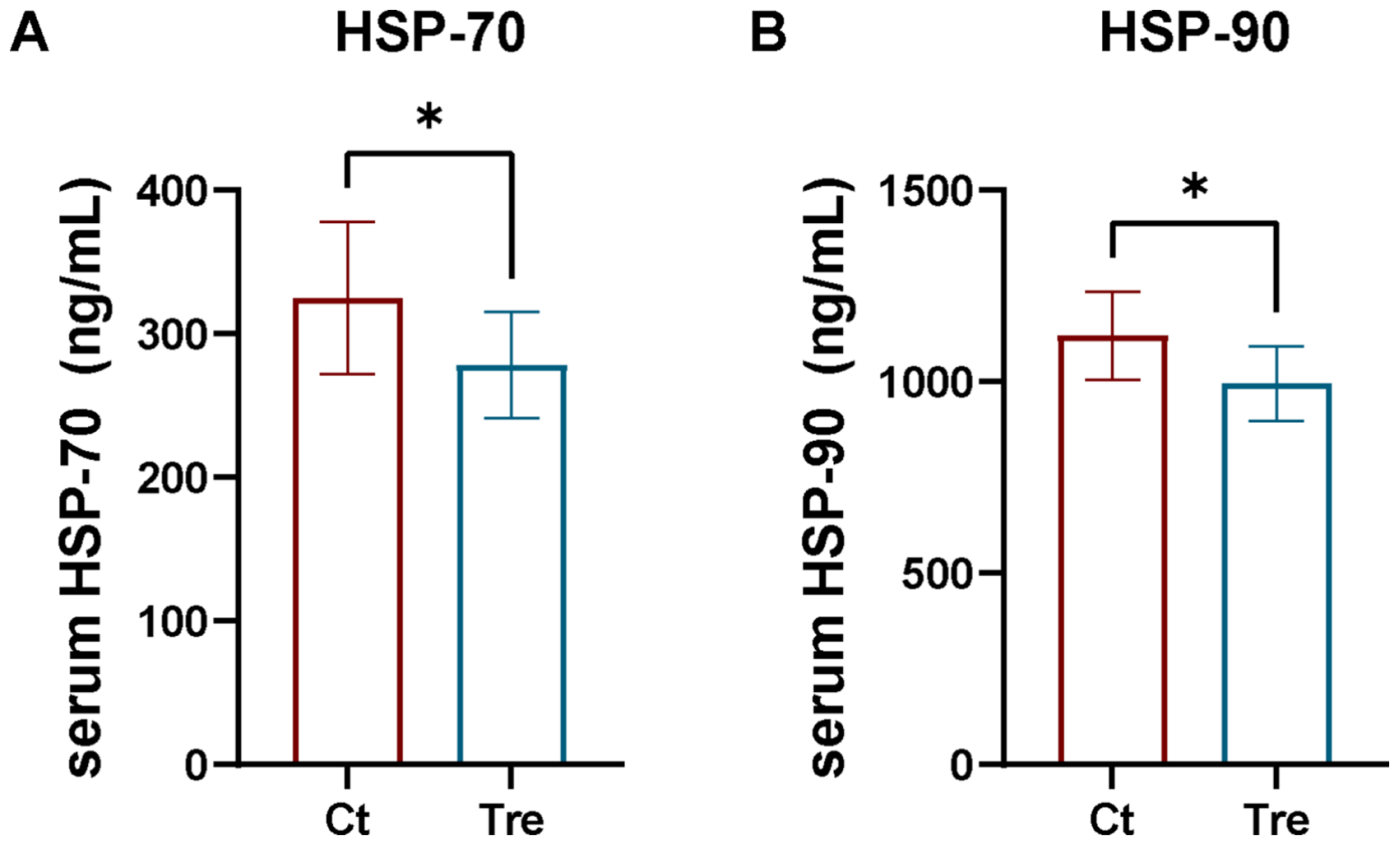
Effects of fermentation bed farming on serum HSP70 **(A)** and HSP90 **(B)** levels in geese following transportation stress. Ct: control group; Tre: fermentation bed group. All data are presented as mean ± SEM. Significance was defined as *p < 0.05, **p < 0.01, and ***p < 0.001 compared to the control group; ‘ns’ indicates not significant.

As shown in Figure 2, geese in the Tre group had significantly lower serum HSP70 and HSP90 levels than those in the Ct group (*p* < 0.05).

### 3.3 Effects of fermentation bed farming on serum stress hormone levels in northern white geese following transportation stress

**Figure 3:**
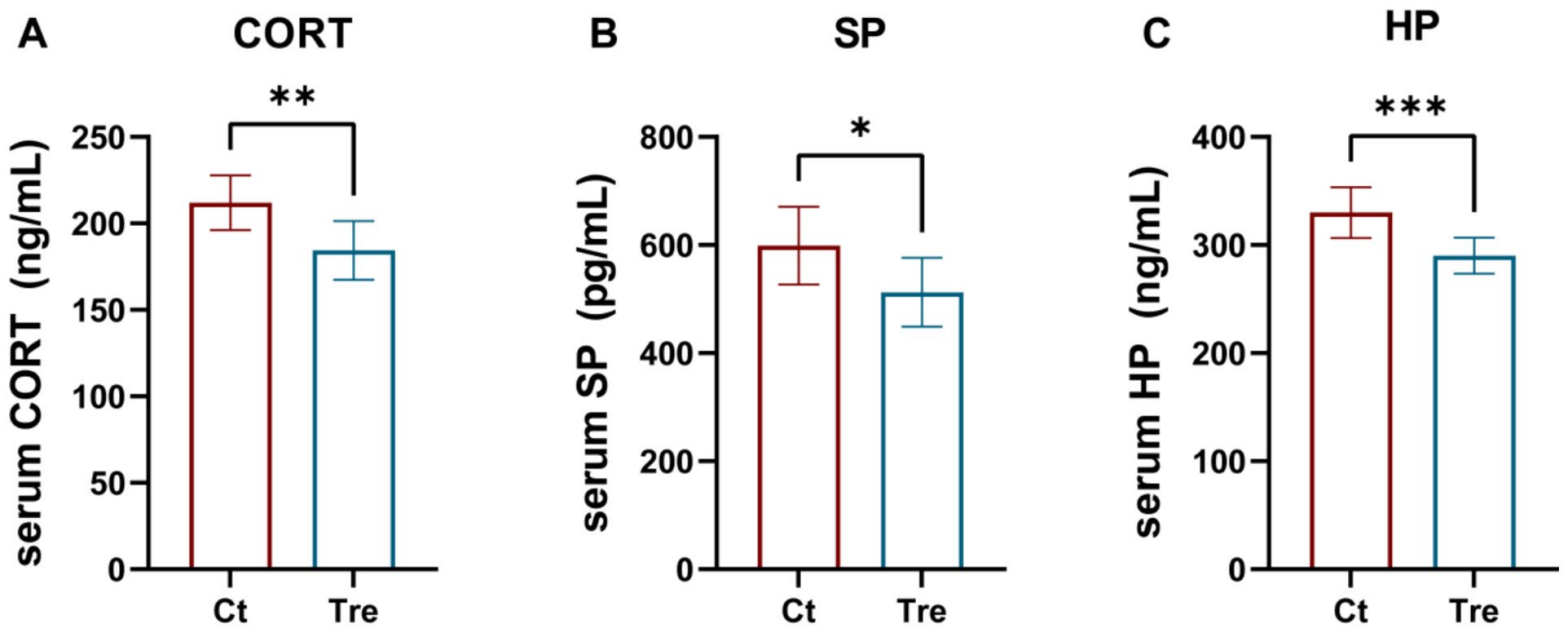
Effects of fermentation bed farming on serum CORT **(A)**, SP **(B)** and HP **(C)** levels in geese following transportation stress. Ct: control group; Tre: fermentation bed group. All data are presented as mean ± SEM. Significance was defined as ^*^p < 0.05, **p < 0.01, and ***p < 0.001 compared to the control group; ‘ns’ indicates not significant.

As shown in Figure 3, geese in the Tre group had significantly lower serum CORT, SP, and HP levels than those in the Ct group (*p* < 0.05).

### 3.4 Effects of fermentation bed farming on serum immunoglobin levels in northern white geese following transportation stress

**Figure 4:**
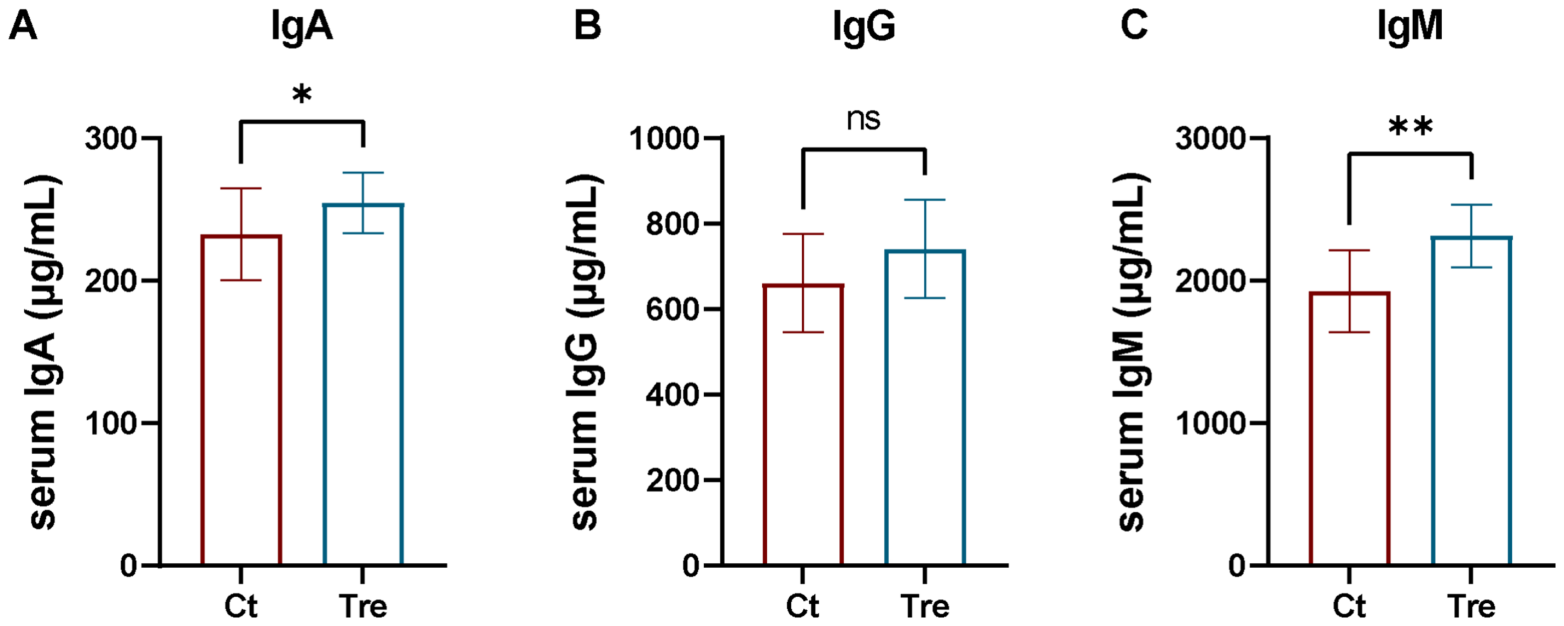
Effects of fermentation bed farming on serum immunoglobin levels in geese following transportation stress **(A–C)**. Ct: control group; Tre: fermentation bed group. All data are presented as mean ± SEM. Significance was defined as *p < 0.05, **p < 0.01, and ***p < 0.001 compared to the control group; ‘ns’ indicates not significant.

As shown in Figure 4, serum IgA and IgM levels were significantly higher in the Tre group than in the Ct group (*p* < 0.05), while no significant difference was found in serum IgG levels between groups (*p* > 0.05).

### 3.5 Effects of fermentation bed farming on serum inflammatory factor levels in northern white geese following transportation stress

**Figure 5:**
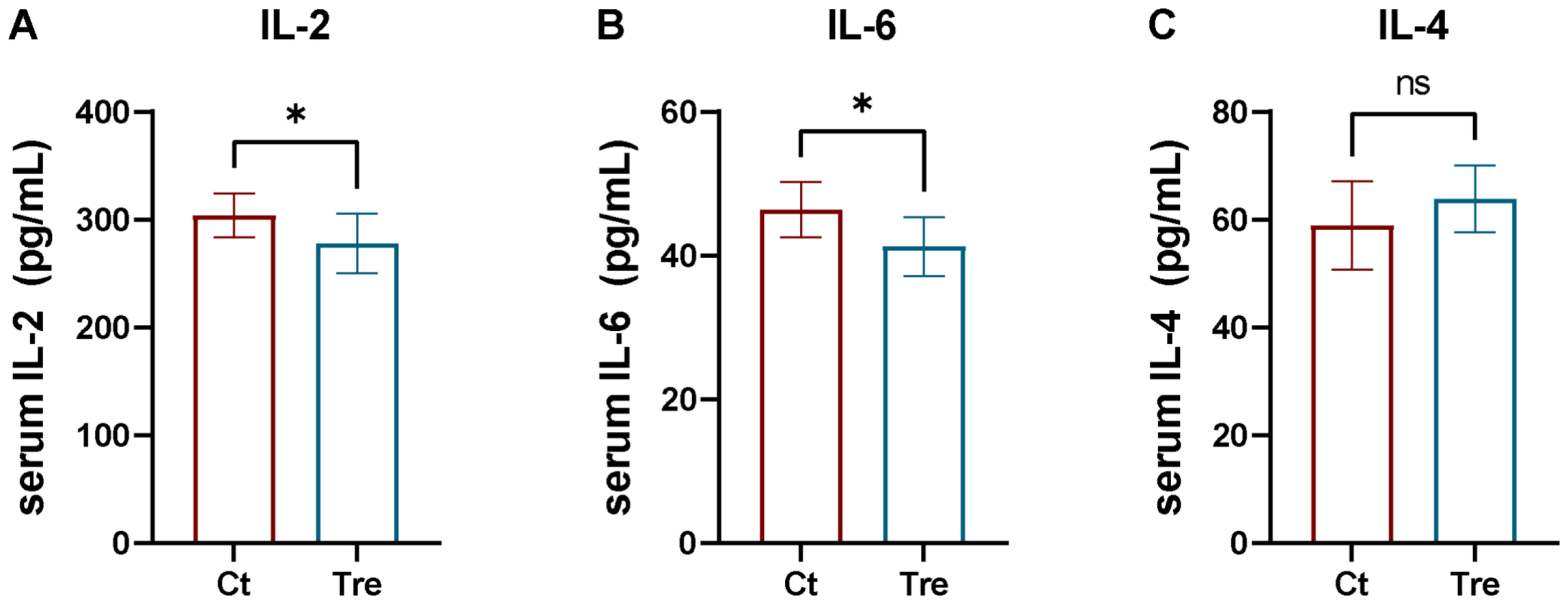
Effects of fermentation bed farming on serum inflammatory factor levels in geese following transportation stress **(A–C)**. Ct: control group; Tre: fermentation bed group. All data are presented as mean ± SEM. Significance was defined as *p < 0.05, **p < 0.01, and ***p < 0.001 compared to the control group; ‘ns’ indicates not significant.

As shown in Figure 5, serum IL-2 and IL-6 levels were significantly lower in the Tre group than in the Ct group (*p* < 0.05), while no significant difference was found in serum IL-4 levels between groups (*p* > 0.05).

### 3.6 Effects of fermentation bed farming on serum antioxidant capacity in northern white geese following transportation stress

**Figure 6:**
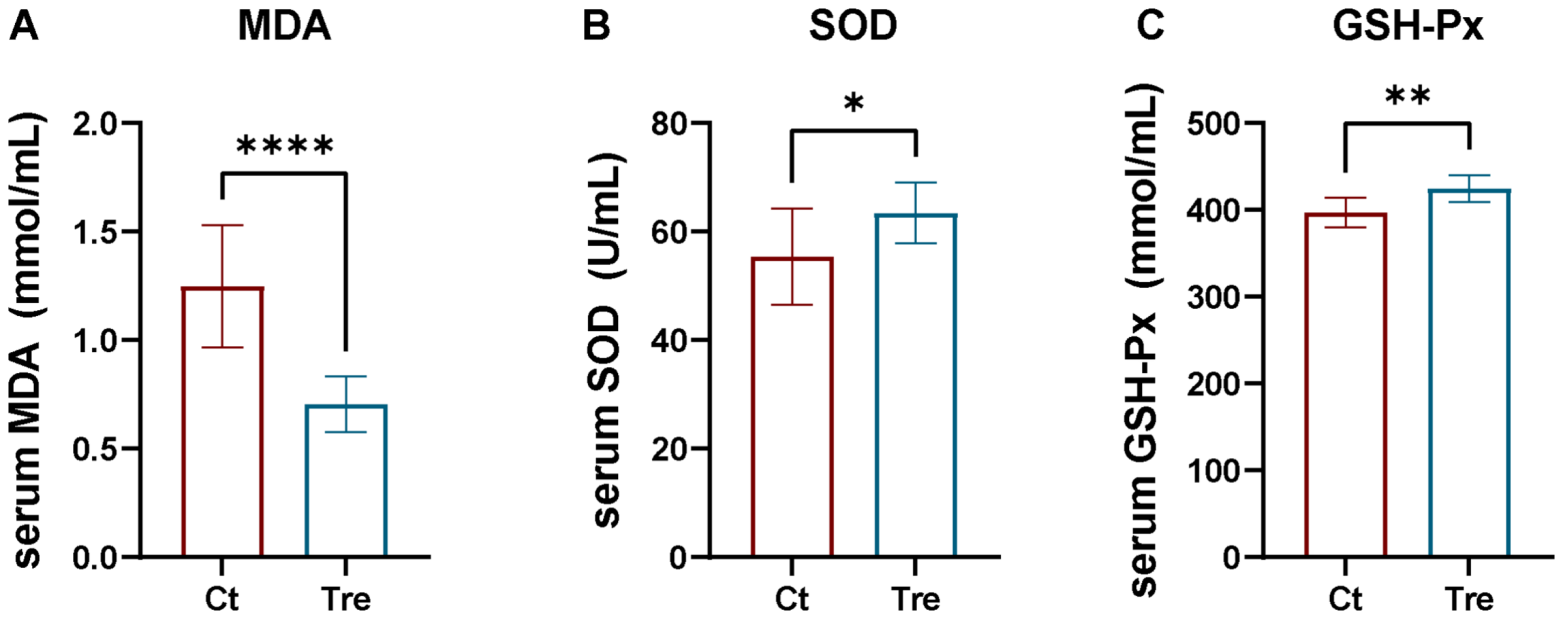
Effects of fermentation bed farming on serum antioxidant capacity in geese following transportation stress **(A–C)**. Ct: control group; Tre: fermentation bed group. All data are presented as mean ± SEM. Significance was defined as *p < 0.05, **p < 0.01, and ***p < 0.001 compared to the control group; ‘ns’ indicates not significant.

As shown in Figure 6, serum SOD and GSH-Px levels were significantly higher in the Tre group than in the Ct group (*p* < 0.05), but the serum MDA level was significantly decreased (*p* < 0.05).

## 4 Discussion

The behavioral expression of poultry serves as a vital indicator reflecting their physiological health status and welfare levels. In this study, fermentation bed farming group significantly increased the duration of lying and walking behaviors in geese while reducing the time spent on standing, may due to the straw which improved the comfort of the ground environment in the rearing facility. The improved comfort increased lying time in geese, while the litter also release stress on their feet, consequently prolonging the duration of walking behavior. Similar research showed laying hens expressed more walking and sitting behaviors in high environmental enrichment rearing environments (18), which also suggests that the fermented bed farming can serve to increase environmental enrichment. Feather pecking represents a significant welfare challenge in poultry production, characterized by birds pulling, pecking, and even consuming each other’s feathers, leading to skin injuries (18, 19). This behavior compromises feather insulation, reduces feed conversion efficiency, and in severe cases, may cause mortality, thereby adversely affecting both animal welfare and productivity (20). This study found that the fermentation bed farming reduced feather pecking but increased foraging pecking behavior in geese, likely because the bedding material provided opportunities for exploration and foraging, thereby redirecting the motivation to peck. Similarly, Pettersson et al. found that providing resource packages, such as additional feeders, metal bells, and shelters to laying hens promoted foraging behavior and reduced feather pecking, effectively preventing skin lesions and open wounds (21, 22). Increased environmental enrichment also encourages more comfort behaviors, such as preening, which was significantly enhanced in hens housed in enriched cages (23). Safe and comfortable conditions further promote behaviors like leg stretching and wing flapping (24). In this study, the bedding material in the fermentation bed system served as a form of environmental enrichment, enabling geese to exhibit a wider range of natural behaviors.

Heat Shock Proteins (HSPs) are highly conserved molecular chaperones and well-established biomarkers of cellular stress. Under normal physiological conditions, HSP expression remains low, but it is significantly upregulated in response to various stressors (e.g., oxidative stress, toxins, thermal stress) to protect cellular integrity and metabolic homeostasis (25–27). Among them, Hsp70 and Hsp90 are core heat shock proteins that are significantly induced and expressed under stress conditions. They participate in protein trafficking and degradation, maintain intracellular protein conformation, protect cells from environmental challenges, and play crucial roles in stress protection (28). Li et al. found that environmental enrichment increased the expression of HSP70 and HSP90 in the spleen of laying hens to enhance stress resistance (16). However, this study showed that serum levels of Hsp70 and Hsp90 were significantly lower in the fermentation bed farming than in the flat-floor farming after experiencing transport stress. The difference maybe due to the enhanced stress resilience developed by geese reared in the enriched fermentation bed environment. Consequently, upon exposure to the same stressor, these geese required a less robust HSP response compared to those raised under flat-floor farming. The hypothalamic–pituitary–adrenal (HPA) axis is activated under stress, leading to elevated glucocorticoid levels (29). Consequently, CORT concentration is a reliable indicator of stress severity in poultry (30). Transport stress is a major challenge in poultry management, known to elevate serum CORT (31). In line with the HSP findings, the fermentation bed group showed significantly lower serum CORT levels post-transport, further supporting the hypothesis that this rearing system enhances stress resistance. Furthermore, SP is a neuropeptide associated with pain thresholds in poultry and serves as a biomarker for pain and nociception in animals (32). Humes et al. demonstrated that SP functions as a key regulator of pain perception and is oversecreted under stressful conditions (33). HP is an acute-phase protein whose concentration has been shown to positively correlate with stress levels in poultry, making it a reliable indicator of stress in animals (34). In this study, serum levels of both substance SP and HP were significantly reduced in geese reared under the fermentation bed farming following transport stress. This synergistic decrease, consistent with the changes in CORT and HSPs, provides multi-dimensional evidence that fermentation bed farming alleviates transport stress by enhancing the overall stress resilience of geese.

Immunoglobulins are vital components of the immune system, maintaining immunological homeostasis. Intense stress can impair immune function and reduce immunoglobulin expression in animals (35). In this study, serum IgA and IgM levels were significantly higher in geese reared under the fermentation bed farming after experiencing transport stress than in those under the flat-floor farming. As environmental enrichment showing increased serum immunoglobulin levels in animals (36), this may explain the increased IgA and IgM concentrations observed in the Tr group. Interleukins (ILs) are key cytokines that modulate immune and inflammatory responses. In particular, IL-2 and IL-6 play pivotal roles in promoting inflammation (37), whereas IL-4, produced by various immune cells, exerts anti-inflammatory functions (38). IL-2 also regulates T lymphocytes and is essential for maintaining immune homeostasis (39). Growing evidence indicates that transport stress triggers the release of various inflammatory factors in poultry (14, 16). In the present study, serum IL-2 and IL-6 levels were significantly lower in the fermentation bed farming after experiencing transport stress, while no significant difference was found in IL-4 levels compared to the flat-floor farming. This suggests that the fermentation bed environment enhanced the stress resilience of geese, thereby suppressing the overexpression of pro-inflammatory cytokines upon acute stress challenge. This finding aligns with previous research by Arranz et al., who reported that environmental enrichment can reduce the expression of inflammatory factors (40), further supporting the anti-inflammatory benefits of the fermentation bed system.

When an organism is subjected to stress, the internal redox balance is disrupted, leading to an imbalance between the production and clearance of free radicals/reactive oxygen species (ROS), thereby causing damage to tissues and cells and resulting in oxidative stress (41). Oxidative stress is one of the major challenges for animals facing during transport (42). To mitigate the harmful effects of oxidative stress, the body produces antioxidant enzymes such as SOD and GSH-Px, which scavenge free radicals and ROS (43). Consequently, the serum levels of SOD and GSH-Px in animals reflect their antioxidant capacity. In this study, serum SOD and GSH-Px levels were significantly higher in geese reared under the fermentation bed system after experiencing transport stress compared to those in the flat-floor farming. This indicates that the fermentation bed farming enhances the antioxidant capacity of geese, thereby reducing damage caused by oxidative stress. MDA is an end product of lipid peroxidation. Its release increases under oxidative stress conditions (44, 45), and MDA levels reflect the extent of lipid oxidation in cells. In this study, serum MDA levels were significantly lower in the fermentation bed group after experiencing transport stress, further revealing that this farming system improves the antioxidant capacity of geese and enhances their overall stress resilience.

In summary, feeding in a fermentation bed farming system improves the behavioral performance of geese and enhances their resistance to transport stress by boosting antioxidant capacity, strengthening immune function, and reducing levels of inflammatory cytokines and stress hormones.

## Data Availability

The original contributions presented in the study are included in the article/supplementary material, further inquiries can be directed to the corresponding authors.
