# Correction: Fermentation bed farming improves behavioral expression and stress resistance in geese

**DOI:** 10.3389/fvets.2026.1823356

**Published:** 2026-03-18

**Authors:** Haoyan Li, Jieyu Yang, Jiexing Zhang, Lingbin Zhao, Tong Zhou, Hongsheng Chen, Jiawei Li, Shuai Zhao, Guoan Yin

**Affiliations:** 1 College of Animal Science and Veterinary Medicine, Heilongjiang Bayi Agricultural University, Daqing, Heilongjiang, China; 2 College of Food Science, Heilongjiang Bayi Agricultural University, Daqing, Heilongjiang, China; 3 College of Informatics, Huazhong Agricultural University, Wuhan, Hubei, China; 4 Heilongjiang Provincial Key Laboratory of Exploration and Innovation Utilization of White Goose Germplasm Resources in Cold Region, Daqing, Heilongjiang, China

**Keywords:** behavior, fermentation bed farming, geese, stress resistance, transport stress

Affiliation Heilongjiang Provincial Key Laboratory of Exploration and Innovation Utilization of White Goose Germplasm Resources in Cold Region, Daqing, Heilongjiang, China was erroneously omitted for authors Shuai Zhao and Guoan Yin.

This affiliation has now been added for author Shuai Zhao and Guoan Yin.

Authors Shuai Zhao and Guoan Yin were erroneously assigned to affiliation College of Informatics, Huazhong Agricultural University, Wuhan, China. This affiliation has now been removed for authors Shuai Zhao and Guoan Yin.

Affiliation College of Animal Science and Veterinary Medicine, Heilongjiang Bayi Agricultural University, Daqing, Heilongjiang, China was erroneously given as Heilongjiang Bayi Agricultural University, Daqing, China.

Affiliation College of Food Science, Heilongjiang Bayi Agricultural University, Daqing, Heilongjiang, China was erroneously given as Northeast Agricultural University, Harbin, China.

Affiliation College of Informatics, Huazhong Agricultural University, Wuhan, Hubei, China was erroneously given as College of Informatics, Huazhong Agricultural University, Wuhan, China.

The original version of this article has been updated.

