# Correction: Fermentation bed farming improves behavioral expression and stress resistance in geese

**DOI:** 10.3389/fvets.2026.1884523

**Published:** 2026-06-02

**Authors:** Haoyan Li, Jieyu Yang, Jiexing Zhang, Lingbin Zhao, Tong Zhou, Hongsheng Chen, Jiawei Li, Shuai Zhao, Guoan Yin

**Affiliations:** 1 College of Animal Science and Veterinary Medicine, Heilongjiang Bayi Agricultural University, Daqing, Heilongjiang, China; 2 College of Food Science, Heilongjiang Bayi Agricultural University, Daqing, Heilongjiang, China; 3 College of Informatics, Huazhong Agricultural University, Wuhan, Hubei, China; 4 Heilongjiang Provincial Key Laboratory of Exploration and Innovation Utilization of White Goose Germplasm Resources in Cold Region, Daqing, Heilongjiang, China

**Keywords:** behavior, fermentation bed farming, geese, stress resistance, transport stress

In the published article, there was a mistake in the Funding statement.

The correct funding statement reads:

## Funding

The author(s) declared that financial support was received for this work and/or its publication. This work was supported by the guiding science and technology plan project of Daqing City (No. zd-2023-69); Heilongjiang Provincial College Students' Innovation Training Program (No. S202410223048); Open Research Fund of Engineering Research Center of Intelligent Technology for Agriculture, Ministry of Education (Grant number ERCITA-KF001).

The original version of this article has been updated.

